# Cloning, Functional Characterization and Response to Cadmium Stress of the Thioredoxin-like Protein 1 Gene from *Phascolosoma esculenta*

**DOI:** 10.3390/ijms23010332

**Published:** 2021-12-29

**Authors:** Jiajie Meng, Xinming Gao, Shengyu Luo, Chenwen Lin, Chen Du, Congcong Hou, Jianping Wang, Shan Jin, Daojun Tang, Chundan Zhang, Junquan Zhu

**Affiliations:** 1 Key Laboratory of Applied Marine Biotechnology by the Ministry of Education, Ningbo University, Ningbo 315012, China; 534603541@139.com (J.M.); nbugxm4851@163.com (X.G.); 1701091031@nbu.edu.cn (S.L.); 1811075010@nbu.edu.cn (C.L.); 8788182@163.com (C.D.); houcongcong@nbu.edu.cn (C.H.); jinshan@nbu.edu.cn (S.J.); tangdaojun@nbu.edu.cn (D.T.); zhangchundan@nbu.edu.cn (C.Z.); 2 Ningbo Academy of Oceanology and Fisheries, Ningbo 315012, China; wjping805@126.com

**Keywords:** thioredoxin-like protein 1, *Phascolosoma esculenta*, cadmium, coelomic fluid

## Abstract

Cadmium (Cd) is a heavy metal toxicant and is widely distributed in aquatic environments. It can cause excessive production of reactive oxygen species (ROS) in the organism, which in turn leads to a series of oxidative damages. Thioredoxin (Trx), a highly conserved disulfide reductase, plays an important role in maintaining the intracellular redox homeostasis in eukaryotes and prokaryotes. *Phascolosoma esculenta* is an edible marine worm, an invertebrate that is extensively found on the mudflats of coastal China. To explore the molecular response of *Trx* in mudflat organisms under Cd stress, we identified a new *Trx* isoform (Trx-like protein 1 gene) from *P. esculenta* for the first time, designated as *PeTrxl*. Molecular and structural characterization, as well as multiple sequence and phylogenetic tree analysis, demonstrated that *Pe*Trxl belongs to the Trx superfamily. *PeTrxl* transcripts were found to be ubiquitous in all tissues, and the highest expression level occurred in the coelomic fluid. Exposure to three sublethal concentrations of Cd resulted in the upregulation and then downregulation of *PeTrxl* expression levels over time in coelomic fluid of *P. esculenta*. The significant elevation of *PeTrxl* expression after 12 and 24 h of Cd exposure at 6 and 96 mg/L, respectively, might reflect its important role in the resistance to Cd stress. Recombinant *Pe*Trxl (*rPe*Trxl) showed prominent dose-dependent insulin-reducing and ABTS free radical-scavenging abilities. After exposure to 96 mg/L Cd for 24 h, the ROS level increased significantly in the coelomic fluid, suggesting that Cd induced oxidative stress in *P. esculenta*. Furthermore, the injection of *rPe*Trxl during Cd exposure significantly reduced the ROS in the coelomic fluid. Our data suggest that *Pe*Trxl has significant antioxidant capacity and can protect *P. esculenta* from Cd-induced oxidative stress.

## 1. Introduction

Cadmium (Cd), an unessential metal element in organisms, is widely distributed in aquatic environments [1]. Current studies have found that the molecular mechanisms of Cd toxicity are diverse, including oxidative damage [2,3], apoptosis [4,5], autophagy [6], DNA damage [7,8] and inhibition of energy metabolism [9,10], with complex relationships existing among them [11]. 

Cd^2+^ in organisms can bind with the sulfhydryl groups of antioxidant enzymes, resulting in their reduced activity or inactivation and indirectly increasing intracellular levels of reactive oxygen species (ROS) [12,13]. Moreover, generating OH radicals via the Fenton reaction between Cd^2+^ and hydrogen peroxide directly leads to an increase in intracellular levels of ROS [14]. The excessive ROS can cause lipid peroxidation of the cell membrane, leading to oxidative damage or apoptosis [15,16]. To minimize the adverse effects of ROS, developing pathways through which excess ROS are scavenged is necessary for organisms. Eukaryotes and prokaryotes have integrated antioxidant systems to resist the harmful effects of ROS, including enzymatic antioxidants (such as thioredoxin (Trx), peroxidase, and catalase) or non-enzymatic antioxidants (such as glutathione (GSH) and vitamin C) [17]. 

The Trx system, composed of Trx, Trx reductase, and NADPH [18], is one of the major antioxidant systems involved in maintaining the intracellular reducing status [19,20,21]. Trx is a small-molecular weight protein (12 kDa) and exists ubiquitously in eukaryotes and prokaryotes [22]. Moreover, its redox active site, a Cys-X_1_-X_2_-Cys (CX_1_X_2_C) motif, plays a reducing role by converting dithiol/disulfide bonds between sulfhydryl groups on two cysteine residues in the active site and the disulfide bond of the target protein [23]. Emerging evidence has shown that Trx plays an important role in DNA replication [24], inhibition of apoptosis [25], and maintenance of cellular redox homeostasis [26]. 

Previous studies have identified many various types of Trx isoforms [27]. Trx-like protein 1 (Trxl; 32 kDa), a member of the Trx superfamily, consists of an N-terminal Trx domain and a C-terminal proteasome-interacting Trx (PITH) domain. However, functional research on Trxl is still in its infancy compared to that for Trx. The Trx domain of Trxl can perform a classical Trx function, repairing oxidatively damaged proteins [28] and scavenging ROS [29] through redox shifts in the CX_1_X_2_C active site. In addition, the PITH domain is a 26S proteasome module is involved in the degradation of intracellular proteins [30]. At present, Trxl has been investigated in various aquatic organisms, including *Apostichopus japonicus* [31], *Hippocampus abdominalis* [32], and *Larimichthys crocea* [33], with particular focus placed on the function of immune stimulation. However, its roles in regulating intracellular redox homeostasis during Cd stress remain unknown in mudflat organisms.

*Phascolosoma esculenta*, belonging to the phylum Sipuncula and the class Phascolosomatidea, is an edible marine invertebrate that resembles a worm and is widely found in the intertidal mudflats of coastal China, living in burrows and feeding on benthic algae and organic debris. As the lives of benthic animals are relatively stable, they are easy to count, and since they are sensitive to environmental changes, they can better reflect the pollution status of water and substrates in the environments of their habitats. Furthermore, *P. esculenta* has a high tolerance to heavy metals [34], and thus, it is a good indicator organism of heavy metal pollution in marine mudflats.

To our knowledge, studies on the response of marine mudflat invertebrate *Trxl* to heavy metals are lacking to date. In the present study, to better understand the role of mudflat organism Trxl in the detoxification of ROS induced by Cd stress, we cloned and characterized a *Trxl* gene (designated as *PeTrxl*) from *P. esculenta*. Furthermore, the tissue distribution and temporal expression profile of *PeTrxl* following exposure to Cd were examined. Finally, *Pe*Trxl was reconstituted in *Escherichia coli* for in vitro activity assays and in vivo functional tests. This study indicated that *Pe*Trxl is involved in the scavenging of ROS induced by Cd and provides important insights into the role of antioxidant systems during heavy metal-induced oxidative stress resistance.

## 2. Results

### 2.1. PeTrxl Full-Length cDNA Cloning and Sequence Analysis 

**Figure 1:**
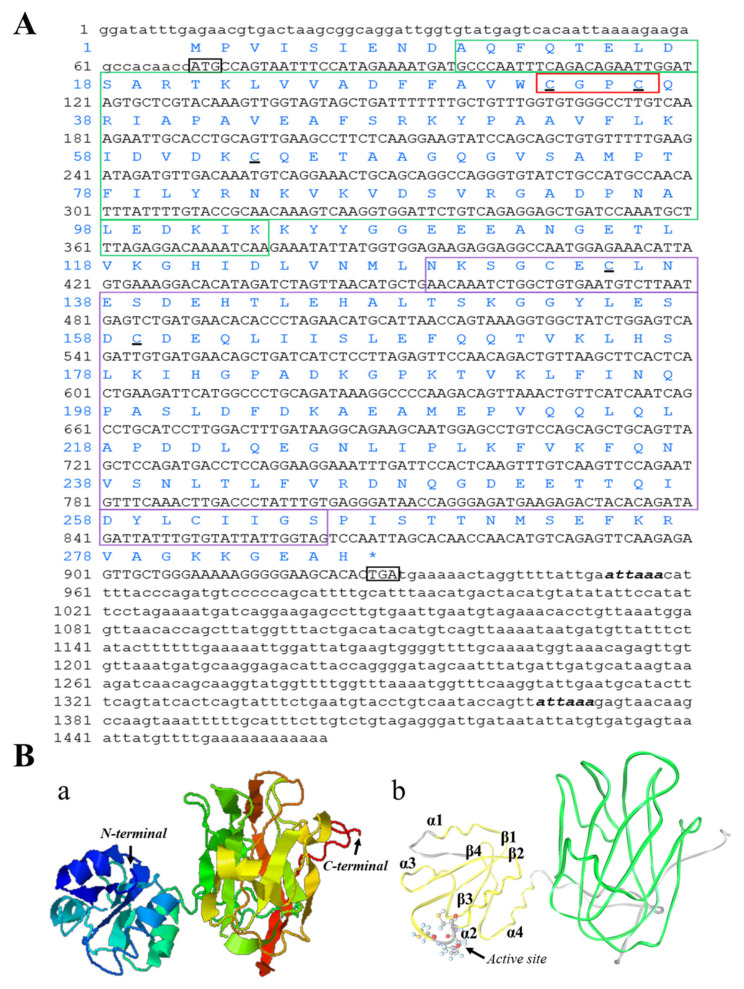
The completed cDNA, deduced amino acid sequence, and the 3D structure of *Pe*Trxl. (**A**) The completed cDNA sequence and deduced amino acid sequence of *PeTrxl*. Start and stop codons are indicated via a black box. Regions indicated by green and purple boxes encode the Trx (10–103 aa) and PITH (129–265 aa) domains, respectively. The catalytic active site ^33^CGPC ^36^ is marked with a red box. The remaining three conserved cysteine residues (Cys_63_, Cys_135_, and Cys_159_) are underlined via a black line. Polyadenylation signals are marked in bold and italics. (**B**) The 3D structure of the *Pe*Trxl protein. (**a**) The N- and C-termini of *Pe*Trxl. (**b**) The yellow and green structures denote the Trx and PITH domains, respectively.

Full-length *PeTrxl* cDNA from *P. esculenta* (GenBank accession No. MW767160) was 1463 bp in length, which included an 861 bp ORF encoding 286 aa, a 69 bp 5′ untranslated region (UTR), and a 533 bp 3′ UTR with two polyadenylation signals (ATTAAA) (Figure 1A). The predicted molecular weight and isoelectric point of the *Pe*Trxl protein were 31.62 kDa and 4.97, respectively. SignalP-5.0 tool analysis revealed that a signal peptide was absent in the *Pe*Trxl sequence. According to the protein subcellular localization prediction, *Pe*Trxl was located in the cytosol and nucleus.

The Cys-Gly-Pro-Cys (CGPC) motif, located at the beginning of the second α-helix, is a typically conserved active site in Trx (Figure 1A,B). The *Pe*Trxl protein sequence structure contained two conserved domains, namely an N-terminal Trx domain (10–103 aa) and a C-terminal PITH domain (129–265 aa; Figure 1A,B). The tertiary structure of the predicted Trx domain was composed of four β-sheets sandwiched by four α-helices, forming an α1β1α2β2α3β3β4α4 structure (Figure 1B). The PITH domain was a general 26S proteasome-interacting module.

### 2.2. PeTrxl Sequence Alignment and Phylogenetic Analysis

**Figure 2:**
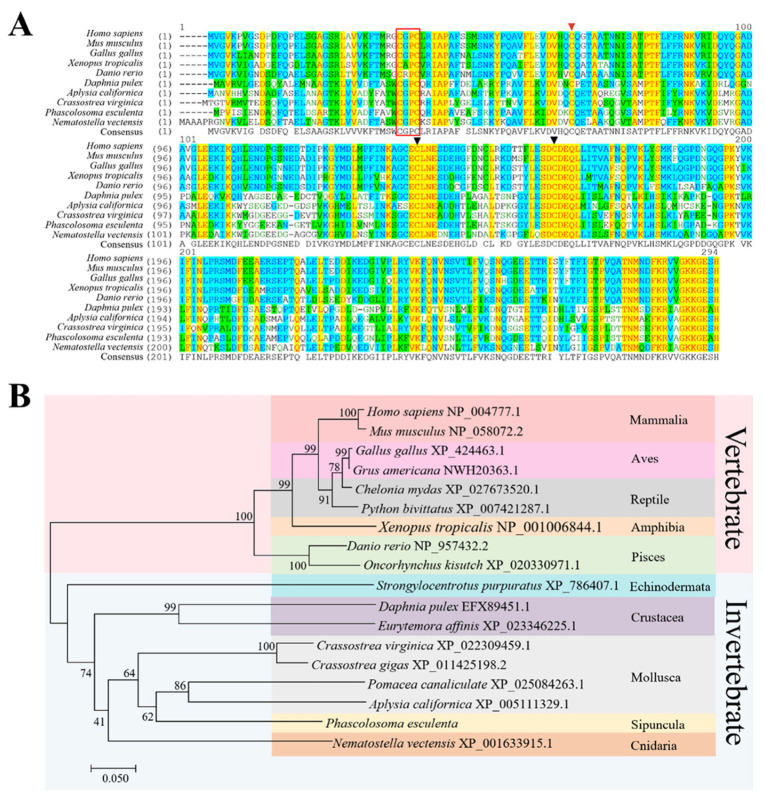
The Multiple sequence alignment and phylogenetic analysis of *Pe*Trxl. (**A**) Multiple sequence alignment of *Pe*Trxl and Trxl amino acid sequences from other species (*H. sapiens* NP_004777.1, 51.2% identity; *M. musculus* NP_058072.2, 51.6% identity; *G. gallus* XP_424463.1, 52.2% identity; *X. tropicalis* NP_001006844.1, 50.2% identity; *D. rerio* NP_957432.2, 50.5% identity; *D. pulex* EFX89451.1, 61.5% identity; *A. californica* XP_005111329.1, 65.5% identity; *C. virginica* XP_022309459.1, 65.6% identity; *N. vectensis* XP_001633915.1, 61.8% identity). The same amino acid residues are shaded in yellow. Blue regions indicate amino acid residues with a similarity greater than 50%; green regions represent lower similarity. The red box indicates the conserved catalytic active site CX_1_X_2_C. Red and black triangles indicate conserved cysteine residues in Trx and PITH domains, respectively. (**B**) Phylogenetic tree analysis of the *Pe*Trxl amino acid sequence. Mega 7.0 software was used to construct a phylogenetic tree; the number at each branch represents the percentage value obtained after 1000 bootstrap replicates were performed. The two branches in the phylogenetic tree are vertebrates, including Mammalia, Aves, Reptilia, Amphibia, and Pisces and invertebrates, including Echinodermata, Crustacea, Mollusca, Sipuncula, and Cnidaria.

The result of the multiple sequence alignment of *Pe*Trxl and Trxl amino acid sequences from other species is shown in Figure 2A; Trxl amino acid sequences of various species are highly homologous. The identities of the *Pe*Trxl amino acid sequence with Trxl amino acid sequences from *Homo sapiens*, *Mus musculus*, *Gallus gallus*, *Xenopus tropicalis*, *Danio rerio*, *Daphnia pulex*, *Aplysia californica*, *Crassostrea virginica*, and *Nematostella vectensis* were 51.2, 51.6, 52.2, 50.2, 50.5, 61.5, 65.5, 65.6, and 61.8%, respectively. The highest homology to *Pe*Trxl was found with that of Mollusca (*C. virginica*, 65.6% identity). As the CX_1_X_2_C motif is a typical redox-active site in the Trx family, it was present in all the aligned sequences. The CGPC motif, which is generally found in the animal kingdom, was observed in *P. esculenta*. In addition, three conserved cysteine residues were found in the Trxl amino acid sequence alignment of the 10 species. The phylogenetic tree constructed using the neighbor-joining tree method showed that Trxl in invertebrates and vertebrates is clustered into two branches (Figure 2B). *Pe*Trxl was clustered with Mollusca Trxl in the invertebrate branch, closest to Trxl from *Pomacea canaliculata* and *A. californica* in the selected species. 

### 2.3. Tissue Distribution of PeTrxl mRNA

**Figure 3:**
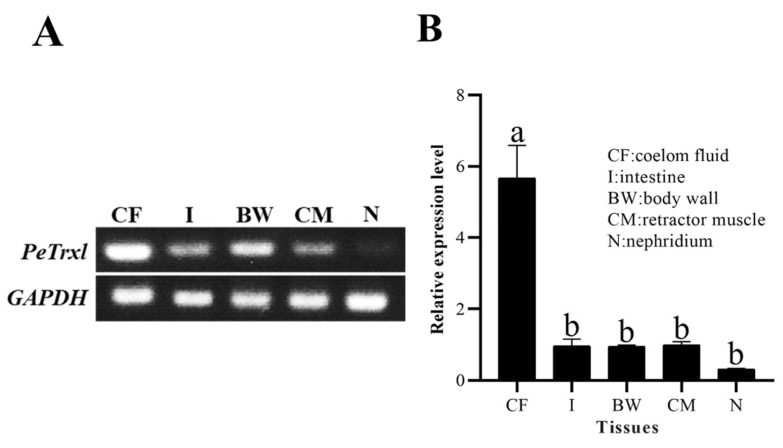
Distribution of *PeTrxl* mRNA in different tissues. (**A**) RT-PCR detection. (**B**) RT-qPCR detection. The *GAPDH* gene was used as an internal reference; data are expressed as the mean ± SD (*n* = 3); different letters indicate significant difference (*p < 0.05*).

Semi-quantitative reverse transcription PCR (RT-PCR) and real-time quantitative PCR (RT-qPCR) were used to detect the transcription levels of *PeTrxl* mRNA in different tissues (Figure 3). As shown in Figure 3, *PeTrxl* mRNA was widely distributed in tissues and could be detected in the coelomic fluid, intestine, body wall, retractor muscle, and nephridium. In addition, the expression level of *PeTrxl* mRNA in the coelomic fluid was significantly higher than that in other tissues, whereas its expression was the lowest in the nephridium. 

### 2.4. PeTrxl mRNA Expression Differences in the Coelomic Fluid following Cd Stress

**Figure 4:**
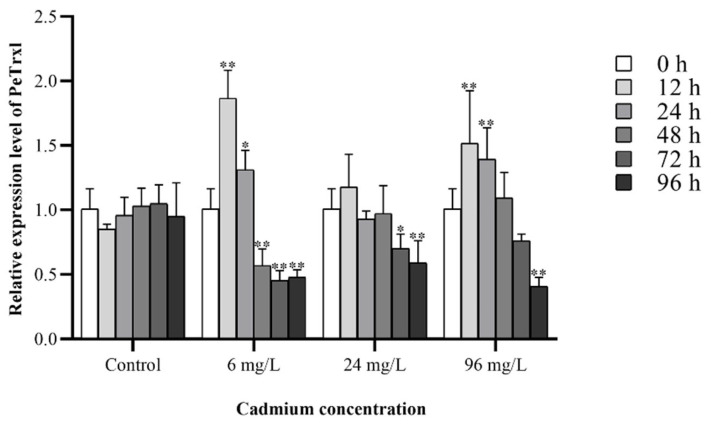
The relative expression level of *PeTrxl* mRNA in the coelomic fluid of *P. esculenta* following Cd stress. Expression characterization of *PeTrxl* mRNA was performed using RT-qPCR. Data are expressed as the mean ± SD (*n* = 4). Significant differences between the Cd-treated group and the control group are shown with an asterisk (* *p* < 0.05; ** *p* < 0.01).

The temporal expression levels of *PeTrxl* mRNA in the coelomic fluid of *P. esculenta*, following exposure to different concentrations of Cd, are shown in Figure 4. *PeTrxl* mRNA expression levels were significantly different in Cd-exposed samples, compared to those in control groups. The control groups maintained a stable expression level of *PeTrxl* throughout the experiment. In contrast, the expression levels of *PeTrxl* increased 1.85- and 1.30-fold after exposure to 6 mg/L of Cd for 12 and 24 h, respectively. Subsequently, *PeTrxl* mRNA levels decreased 1.78-, 2.22-, and 2.11-fold after 48, 72, and 96 h, respectively. *PeTrxl* mRNA levels decreased by 1.44- and 1.71-fold after 72 and 96 h, respectively, following exposure to 24 mg/L of Cd. No significant change in *PeTrxl* mRNA levels was observed after 12, 24, and 48 h of exposure to 24 mg/L of Cd. Following exposure to Cd levels under 96 mg/L, *PeTrxl* mRNA expression levels increased 1.5- and 1.38-fold at 12 and 24 h, respectively, and decreased 2.47-fold at 96 h. *PeTrxl* mRNA levels peaked 12 h after exposure to 6 mg/L of Cd.

### 2.5. PeTrxl Expression and Purification

**Figure 5:**
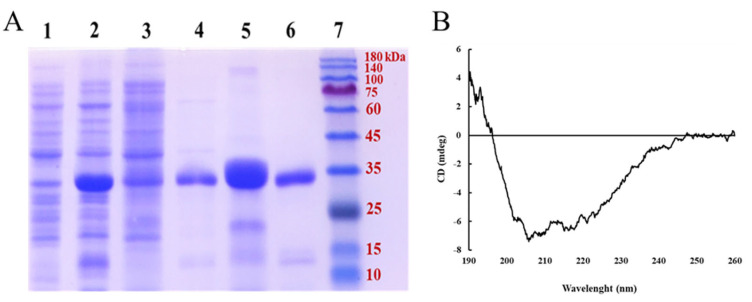
(**A**) SDS-PAGE analysis of recombinant *Pe*Trxl. *rPe*Trxl was separated using 10% SDS-PAGE; protein bands were visualized after staining with Coomassie Brilliant Blue R250. Line 1: lysate of *E. coli* (pET28a-*PeTrxl*) without induction; Line 2: lysate of *E. coli* (pET28a-*PeTrxl*) induced by IPTG; Line 3: supernatant of lysate; Line 4: pellet of lysate; Line 5: purified *rPe*Trxl; Line 6: refolded *rPe*Trxl; Line 7: protein marker. (**B**) Circular dichroism spectral analysis of refolded *rPe*Trxl.

As shown in Figure 5A, we transfected *E. coli* cells with the *PeTrxl* gene, also successfully inducing its expression. SDS-PAGE (sodium dodecyl sulfate polyacrylamide gel electrophoresis) analysis revealed that the recombinant *Pe*Trxl (*rPe*Trxl) was present in the inclusion bodies. A single band of purified *rPe*Trxl was approximately 31.62 kDa, which was consistent with the theoretical value predicted by the ProtParam tool. Next, purified *rPe*Trxl was renatured using the dialysis refolding method; the concentration of refolded *rPe*Trxl was 956 μg/mL, as measured using Bradford’s method. 

The results of the circular dichroism spectroscopy indicated that the refolded *rPe*Trxl has a significant secondary structure absorption spectrum (Figure 5B). The percentage of secondary structure is 13.5% α-helices, 39.5% β-sheets, 8.9% β-turns, and 38.1% random coils (Table 1). 

### 2.6. In Vitro Refolded rPeTrxl Activity Assay

#### 2.6.1. Insulin Disulfide Reduction Assay

**Figure 6:**
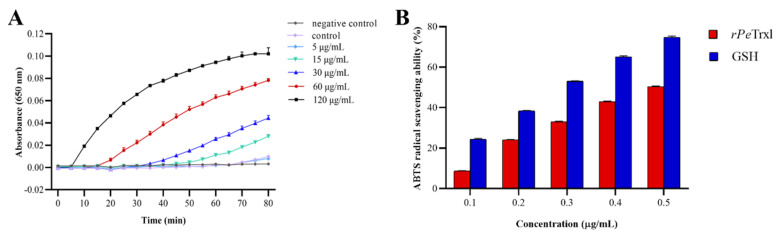
*rPe*Trxl redox activity detection. (**A**) *rPe*Trxl insulin disulfide reduction assay. The final concentrations of *rPe*Trxl in the reaction mixture were 0, 5, 15, 30, 60, and 120 μg/mL. The absorbance (Ab; 650 nm) of the reaction mixture was monitored following the addition of DTT. The reaction system without DTT and *rPe*Trxl was the negative control. Data are presented as the mean ± SD (*n* = 3). (**B**) ABTS radical scavenging assay of *rPe*Trxl. Different concentrations of GSH (0.1, 0.2, 0.3, 0.4, and 0.5 μg/mL) were used as the positive control. Data are presented as the mean ± SD (*n* = 3).

The specific reducing activity of *rPe*Trxl was investigated using an insulin disulfide reduction assay (Figure 6A). The results showed that in the presence of dithiothreitol (DTT), the disulfide bonds between the A and B chains of insulin were broken in the experimental group with *rPe*Trxl, with the precipitation caused by B chain aggregation increasing the absorbance (Ab) of the reaction system at 650 nm. In addition, increasing the concentration and reaction time resulted in significant increases in the Ab, indicating that the reducing activity of *rPe*Trxl was time- and dose-dependent. In contrast, owing to a lack of *rPe*Trxl, the Ab increased only slightly in the control group during the experiment; the negative control group did not exhibit a change in Ab_650_.

#### 2.6.2. ABTS Radical-Scavenging Assay

The ABTS (2,2′-azino-bis(3-ethylbenzothiazoline-6-sulfonic acid)) radical is a stable organic radical. The ABTS radical-scavenging rate of *rPe*Trxl corresponded to its antioxidant capacity. As shown in Figure 6B, the ABTS radical scavenging rates of *rPe*Trxl and GSH were positively correlated with their concentrations. Both *rPe*Trxl and GSH had the highest ABTS radical-scavenging rates at 0.5 mg/mL, specifically 50.35 and 74.71%, respectively, at which point, the gap between them was also the smallest. In addition, the IC_50_ of the ABTS radical-scavenging ability of *rPe*Trxl and GSH was 0.488 and 0.255 mg/mL, respectively; higher IC_50_ values indicated a lower ABTS radical-scavenging ability. These results illustrate that *rPe*Trxl has a high ability to scavenge ABTS radicals.

### 2.7. In Vivo Refolded rPeTrxl Activity Assay

**Figure 7:**
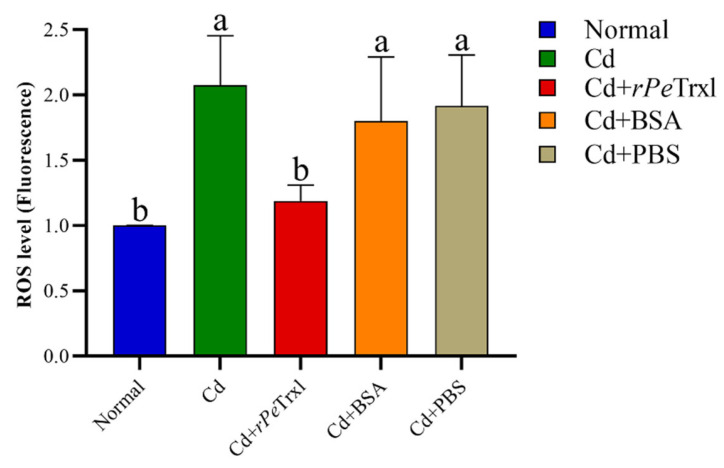
In vivo reactive oxygen species (ROS) level variation in *P. esculenta* following *rPe*Trxl injection. Flow cytometry was used to monitor the fluorescence intensity of ROS in coelomic fluid cells. The normal group, without exposure to Cd, served as the blank control, whereas the Cd-treated group was the positive control. Cd exposure groups injected with BSA and PBS, respectively, served as control groups. Significant differences are indicated via different letters (*p* < 0.05). Data are presented as the mean ± SD (*n* = 9).

To further investigate the redox function of *rPe*Trxl under Cd stress, it was injected into *P. esculenta*. As shown in Figure 7, ROS levels in the coelomic fluid of *P. esculenta* were significantly increased in the Cd-treated group and the Cd-treated groups injected with BSA and PBS, respectively, compared with those in the blank control and *rPe*Trxl-injection groups. However, no significant differences were observed in ROS levels between the blank control and *rPe*Trxl-injection groups.

## 3. Discussion

### 3.1. Characterization of PeTrxl

This is the first study to clone and identify the complete cDNA sequence of *PeTrxl* from *P. esculenta*. The CGPC motif, a typical redox active site belonging to Trx, was found in the *Pe*Trxl amino acid sequence (Figure 1A), which is consistent with Trxl from *H. sapiens* [35], *A. japonicus* [31], and *Strongyloides Ratti* [36]. Our results indicated that *Pe*Trxl contained two conserved domains, namely an N-terminal Trx domain and a C-terminal PITH domain (Figure 1A). The structure of the Trx domain consists of four β-sheets surrounded by four α-helices [32,37,38], in the formation of α1β1α2β2α3β3β4α4 (Figure 1B). The active site (CGPC) motif of the Trx domain, located at the beginning of the second α-helix, maintains intracellular redox homeostasis via the exchange reaction of thiol/disulfide bonds between two cysteine residues [23]. In addition, the PITH domain, a general 26S proteasome-interacting module, is able to bind to 19S regulatory complexes (the components of the 26S proteasome) in mammals to participate in protein deubiquitination and degradation [30,39,40]; this domain exists in all Trxl proteins [36]. The amino acid sequence of Trxl is relatively evolutionarily conserved. Multiple sequence alignment indicated that *Pe*Trxl possessed the highest similarity (65.6%) with Trx1 of *C. virginica*, whereas similarities with those of all species in Figure 2A ranged from 50 to 65.6%. Meanwhile, phylogenetic tree analysis revealed that *Pe*Trxl had the highest homology with Mollusca (*P. canaliculata* and *A. californica*) Trxl (Figure 2B). Based on these results, we identified *Pe*Trxl as a new member of the Trx superfamily.

### 3.2. Tissue Distribution of PeTrxl and Its Expression during Cd Stress

Cd is generally a peroxide inducer that causes oxidative damage to cells by binding to the thiol groups of enzymes, thereby causing a change in the enzyme spatial conformation and leading to a decrease in enzyme activity [12,41,42]. To resist the oxidative damage caused by ROS, organisms have developed a complete antioxidant system [17]. Trx is an important antioxidant in organisms [19]. At present, only a few reports have investigated variations in *Trx* gene expression following Cd exposure in aquatic animals. In this study, the tissue distribution of *PeTrxl*, as well as its response to different Cd concentrations and exposure times, was investigated.

Extensive research has revealed that *Trxl* is abundant, stable, and ubiquitously expressed in the cells of all examined vertebrate and invertebrate tissues [30,31,33,43]. Similarly, *PeTrxl* mRNA was detected in all tissues examined in this study, suggesting that this gene might be involved in an important physiological function. However, tissues with high expression levels of *Trxl* mRNA were determined to be different in various species. For example, in the big-belly seahorse *H. abdominalis* [32] and the Chinese honeybee *Apis cerana cerana* [44], the highest expression levels of *Trxl* mRNA were found in the muscle and epidermis, respectively. In *P. esculenta*, the highest and lowest *PeTrxl* mRNA expression levels were detected in the coelomic fluid and nephridium, respectively. Coelomic fluid, mainly consisting of blood cells, granular cells, and germ cells [45], accounts for approximately half of the body weight of *P. esculenta* and is important for immunity and energy metabolism [46,47,48]; thus, the expression level of *PeTrxl* in coelomic fluid during Cd stress was examined. Previous studies have demonstrated that low concentrations and short periods of Cd stress can activate the antioxidant system in organisms, enhancing the scavenging capacity for ROS; however, high concentrations and long periods of Cd stress exceed the detoxification capacity of the antioxidant system, thereby causing oxidative damage [7,49,50]. In our study, we found that *PeTrxl* mRNA expression levels correlated with the concentration and duration of Cd exposure (Figure 4), showing significant upregulation in both the low and high concentration (6 mg/mL and 96 mg/mL) groups after 12 and 24 h of Cd exposure and indicating that *PeTrxl* mRNA is sensitive to Cd stress in the early stages. *PeTrxl* mRNA expression was upregulated to reduce and repair oxidized or damaged proteins [31,32]; similar phenomena have been reported in previous studies [51,52,53]. However, *PeTrxl* mRNA expression levels were significantly downregulated in the three Cd concentration groups, compared with those in the control group, after 96 h of Cd exposure, potentially explaining the antioxidant system damage caused by Cd accumulation over time [7]. Alternatively, the expression level of *PeTrxl* began to decrease gradually after 12 h, which may be related to the activation of other antioxidant and detoxification genes [52,54,55]. These genes have important functions in the maintenance of redox homeostasis, resulting in a weakened requirement for *PeTrxl*. It is worth mentioning that the expression level of *PeTrxl* did not change significantly at 12 and 24 h under moderate stress (24 mg/mL). Although the exact cause remains unclear, we speculate that *PeTrxl* may be an extremely Cd-sensitive gene that is activated and involved in scavenging ROS before 12 h, after which its expression level begins to decrease with the activation of other antioxidant systems. These results suggest that *PeTrxl* is an important functional gene and involved in the detoxification of *P. esculenta* at the early stages of Cd stress. Additionally, *PeTrxl* can be used as a biomarker to study the detoxification mechanism of invertebrate mudflat organisms under heavy metal exposure.

### 3.3. Function of PeTrxl in Cd Stress

Trx, an important functional protein, maintains cellular redox homeostasis and has multiple intracellular and extracellular functions [56,57]. At present, in aquatic animals, many studies have focused on investigating the Trx function associated with antibacterial and antiviral immunity; few studies have reported the function of Trx in resisting heavy metal stress. In this study, the activity of *rPe*Trxl was detected in vitro, and its function in Cd stress was further examined.

In our study, the activity of *rPe*Trxl was investigated using insulin disulfide reduction [58] and ABTS radical scavenging assays. The results of the former assay showed that insulin was reduced by *rPe*Trxl, with the Ab then increasing at 650 nm as precipitation proceeded (Figure 6A). In addition, the reduction efficiency of *rPe*Trxl increased with time in a dose-dependent manner, a pattern demonstrated in previous studies [31,33,36]. However, a slight change in Ab was observed in the control group during the experiment. Interestingly, a similar result was detected in *Haliotis discus discus* [59], *Trichoderma reesei* [60], and *Spodoptera litura* [61]. This might be a result of the slow reduction of insulin mediated by DTT. The ABTS radical scavenging assay is typically used to detect the antioxidant capacity of antioxidants. Our results showed that the ABTS radical scavenging efficiency of *rPe*Trxl increased in a dose-dependent manner and was lower than that of GSH (Figure 6B). Previous studies have revealed that Trx can effectively scavenge hydroxyl and DPPH free radicals [32,62]. Based on these results, we infer that *Pe*Trxl is a protein with antioxidant activity that might perform its function by either reducing disulfide bonds in proteins or reacting directly with ROS.

Moreover, to further investigate the function of *Pe*Trxl, the role of *rPe*Trxl in Cd stress was investigated, for the first time, by injecting the recombinant protein in vivo. The results showed that levels of ROS in the coelomic fluid cells of *P. esculenta* were significantly upregulated after 24 h of Cd stress (96 mg/L); however, this level was significantly lower in the group injected with *rPe*Trxl than in the other experimental groups (Figure 7). This difference could be caused by the involvement of *rPe*Trxl in the scavenging of ROS and demonstrate that *Pe*Trxl can perform its antioxidant function in the extracellular space. Notably, although Trx lacks a signal peptide, it can be secreted into the extracellular compartment via a non-classical pathway [63] and protect cells in response to oxidative stress and inflammation [64]. Similar to Trx, Trxl can also be released into the extracellular space to exert its effects [36]. These results suggest that *Pe*Trxl is a powerful antioxidant protein that can effectively scavenge ROS. Additionally, the injection of *rPe*Trxl may be an effective way of antioxidant.

## 4. Materials and Methods

### 4.1. Experimental Animals, Treatments, and Sampling

Healthy *P. esculenta* (3.9 ± 0.7 g) were obtained from Xizhou of Ningbo (Zhejiang, China); they were acclimatized in 32 cm × 21 cm × 20 cm constantly aerated plastic tanks with 28‰ filtered seawater at 22 ± 0.5 °C for 24 h. Individuals were randomly and evenly distributed across 12 tanks, and a CdCl_2_ solution was prepared with CdCl_2_·2.5H_2_O (Sinopharm, Shanghai, China). Four Cd-exposure concentrations, including 0, 6, 24, and 96 mg/L, corresponding to 0, 1/32, 1/8, and 1/2 of the LC_50_ value, respectively, based on our previous studies (pre-test) investigating the effect of Cd^2+^ exposure on *P. esculenta*, were used in the toxicity experiment. Three replicate tanks were used for each treatment. Following 0, 12, 24, 48, 72, and 96 h of exposure, coelomic fluids (mainly including blood cells, granular cells, and germ cells) were collected from six randomly selected individuals treated with each concentration. The coelomic fluid, body wall, intestine, retractor muscle, and nephridium were sampled for tissue distribution detection. All samples were immediately frozen in liquid nitrogen upon dissection and stored at −80 °C. All experimental procedures were approved by the Animal Care and Use Committee of Ningbo University (Ningbo, China).

### 4.2. Full-Length cDNA Cloning of PeTrxl

First, we obtained a partial cDNA sequence (GenBank accession No. OL757513) of *PeTrxl* from transcriptome data. Next, specific primers (Table 2) were designed using Primer Premier 5.0 software, and the cDNA sequence was verified by sequencing the ORF fragment. Touchdown PCR parameters were as follows: 94 °C for 5 min; 10 cycles of 94 °C for 30 s, 59 °C for 30 s (decreased by 0.5 °C/cycle), and 72 °C for 50 s; 30 cycles of 94 °C for 30 s, 54 °C for 30 s, and 72 °C for 45 s; and a final extension at 72 °C for 10 min. PCR products were separated on 1% agarose gels, and the target bands were obtained by purifying the gel products with a DNA Gel Extraction Kit (BioTeke, Beijing, China). Next, purified products were ligated into the PMD-18T vector and transformed into competent cells (*E. coli* DH5α). Positive clones were identified using PCR and M13F/R primers and subsequently sequenced by GENEWIZ (Suzhou, Jiangsu, China).

Based on the aforementioned intermediate fragment, 5′ and 3′ RACE primers (Table 2) were designed for the rapid amplification of cDNA ends (RACE). Touchdown PCR reaction conditions for 5′ and 3′ RACE were as follows: 94 °C for 5 min; 8 cycles at 94 °C for 30 s, 69 °C/63 °C for 30 s (decreased by 0.5 °C/cycle), and 72 °C for 40 s/1 min; 30 cycles at 94 °C for 30 s, 65 °C/59 °C for 30 s, and 72 °C for 40 s/1 min; followed by 10 min at 72 °C for the final extension. Processing and sequencing of the PCR products were consistent with the aforementioned methods.

### 4.3. Sequence Analysis

The ORF was predicted using the NCBI online tool ORFfinder (http://www.ncbi.nlm.nih.gov/Structure/cdd/wrpsb.cgi, accessed 20 April 2021), whereas protein translation was evaluated using the Sequence Manipulation Suite (http://www.bio-soft.net/sms/, accessed 10 May 2021). The conserved domain of *Pe*Trxl was analyzed using the NCBI conserved domain search (http://www.ncbi.nlm.nih.gov/Structure/cdd/wrpsb.cgi, accessed 10 May 2021). The molecular weight and isoelectric point were calculated using the ProtParam tool (http://web.expasy.org/protparam/, accessed 10 May 2021). Potential signal peptides were determined using the SignalP-5.0 server (http://www.cbs.dtu.dk/services/SignalP/, accessed 10 May 2021). Secondary structure prediction was performed using the NPS@ server (https://npsa-prabi.ibcp.fr/, accessed 10 May 2021), and the 3D structure of *Pe*Trxl was built using I-TASSER (https://zhanglab.ccmb.med.umich.edu/I-TASSER/, accessed 10 May 2021). *Pe*Trxl protein subcellular localization was predicted using WoLF PSORT (https://wolfpsort.hgc.jp/, accessed 10 May 2021). Vector NTI 11.5 (Invitrogen, CA, USA) and Mega 7 (Informar Technologies, Los Angeles, CA, USA) software were used for protein multiple sequence alignment and construction of the amino acid sequence phylogenetic tree, respectively. The amino acid sequences of the Trxl protein discussed in our study were downloaded from NCBI; these included those of *H. sapiens* (NP_004777.1), *M. musculus* (NP_058072.2), *G. gallus* (XP_424463.1), *Grus americana* (NWH20363.1), *Chelonia mydas* (XP_027673520.1), *Python bivittatus* (XP_007421287.1), *X. tropicalis* (NP_001006844.1), *D. rerio* (NP_957432.2), *Oncorhynchus kisutch* (XP_020330971.1), *Strongylocentrotus purpuratus* (XP_786407.1), *D. pulex* (EFX89451.1), *Eurytemora affinis* (XP_023346225.1), *C. virginica* (XP_022309459.1), *Crassostrea gigas* (XP_011425198.2), *A. californica* (XP_005111329.1), *P. canaliculata* (XP_025084263.1), and *N. vectensis* (XP_001633915.1). 

### 4.4. Tissue Distribution and Expression Differences

RT-PCR and RT-qPCR were performed to analyze the distribution of *PeTrxl* in different tissues. Its distribution in the coelomic fluid, intestine, body wall, retractor muscle, and nephridium was detected using a RT-PCR program (pre-denaturation at 94 °C for 5 min; 30 cycles at 94 °C for 30 s, 59 °C for 30 s, 72 °C for 30 s; final extension at 72 °C for 10 min). PCR products were separated using 1% agarose gel electrophoresis; visual images were obtained using a gel image analysis system (FR, Shanghai, China). The RT-qPCR assay was conducted using the LightCycler480 II instrument (Roche, Basel, Basel-Stadt, Switzerland) to detect *PeTrxl* tissue distribution and expression differences. The 20 µL reaction system contained 5 µL of diluted cDNA, 10 µL of 2× RealStar Green Fast Mixture (GenStar, Beijing, China), 3 µL of ddH_2_O, and 1 µL of each primer. The RT-qPCR assay program was as follows: 95 °C for 5 min and 40 amplification cycles (95 °C for 15 s, 59 °C for 15 s, 72 °C for 15 s, and 72 °C for 1 s to collect the fluorescence signal). The *GAPDH* gene was used as the internal control, based on Su et al. [65]. The RT-qPCR primers used are listed in Table 2. The comparative 2^−ΔΔ*C*t^ method was used to analyze the relative expression levels of *PeTrxl* mRNA, presented as the mean ± standard deviation (SD). Significant differences (*p* < 0.05) were analyzed using one-way ANOVA (with least significance difference (LSD) post hoc tests and Duncan post hoc tests) with SPSS 20.0 software (IBM Corporation, Armonk, NY, USA).

### 4.5. Recombinant Plasmid Construction and Protein Expression

The *PeTrxl*-coding region was amplified using specific primers with restriction sites (*Bam*HI and *Xho*I) (Table 2). After digestion with *BamH I* and *Xho I* (Thermo Fisher Scientific, Waltham, MA, USA), the PCR product was ligated with a pET28a (+) vector (Solarbio, Beijing, China) digested using the same method. The recombinant plasmid was transformed into Transetta(DE3) (TransGen Biotech, Beijing, China), and positive transformants were selected and sequenced. After ensuring insertion of the coding fragment, cells were cultured in liquid medium (+Kanamycin) at 37 °C with shaking at 220 rpm. When the OD_600_ value of the bacterial solution was 0.4–0.8, isopropylthiogalactoside (IPTG; Solarbio, Beijing, China) was added at a final concentration of 1 mM and cells were cultured at 37 °C and 220 rpm for 8 h to induce *Pe*Trxl expression.

### 4.6. Purification and Renaturation of Recombinant PeTrxl Protein

Following centrifugation of the induced bacterial solution, target cells were harvested and purified using HisTrap Ni-Agarose Resin (Cowin Biotech, Beijing, China), according to the manufacturer’s instructions. In addition, Triton X-100 (Solarbio, Beijing, China) was used to remove the endotoxin. Recombinant *Pe*Trxl (*rPe*Trxl) was refolded via dialysis renaturation. Briefly, MD34 (8000–14,000 D) dialysis membranes (Solarbio, Beijing, China) were used to dialyze the recombinant protein in a 50 mM phosphate-buffered saline (PBS) solution containing 6, 4, 3, 2, 1, and 0 M (three times) urea (Solarbio, Beijing, China) for 12 h in sequence. In addition, all concentrations of urea, excluding 0 M, were supplemented with 10% glycerin (Solarbio, Beijing, China), 2 mM GSH (Solarbio, Beijing, China), and 0.02 mM GSSG (L-GSH oxidized; Solarbio, Beijing, China) to improve refolding efficiency in the dialysate. Refolded *rPe*Trxl was concentrated using poly(ethylene glycol) 20,000 (Solarbio, Beijing, China). Finally, the Bradford protein assay kit (Beyotime, Shanghai, China) was used to measure the protein concentration; protein bands were separated using 10% SDS-PAGE. Visible protein bands were obtained by staining with Coomassie Brilliant Blue R250 (Solarbio, Beijing, China).

In order to investigate the refolding efficiency of *rPe*Trxl, circular dichroism measurement was performed by Jasco J-1500 spectropolarimeter (Jasco, Tokyo, Japan) with 1 mm path length cuvette at 20 °C. The *rPe*Trxl was scanned at the wavelength range of 190–260 nm with a speed of 100 nm/min to collect circular dichroism spectral data. The data integration time was set to 1 s, data pitch to 0.1 nm, bandwidth to 1 nm. Scan each sample three times and take the average value as the final result. The secondary structure elements of *rPe*Trxl were estimated using SpectraManager software (Jasco, Tokyo, Japan).

### 4.7. In Vitro Refolded rPeTrxl Activity Assay

#### 4.7.1. Insulin Disulfide Reduction Assay

The antioxidant activity of refolded *rPe*Trxl was detected using an insulin disulfide reduction assay (Holmgren, 1979), in which 0.6 mL of the reaction mixture was composed of 402 µL of 50 mM PBS (pH 7.4), 100 µL of 2 mg/mL insulin (bovine; Solarbio, Beijing, China), 12 µL of 10 mM EDTA (Solarbio, Beijing, China), 6 µL of 100 mM DTT (Solarbio, Beijing, China), and 80 µL of different final concentrations of refolded *rPe*Trxl, including 5, 15, 30, 60, and 120 μg/mL. All drugs were dissolved in 50 mM PBS. For the control, refolded *rPe*Trxl was replaced with an equal volume of 50 mM PBS; the negative control was the solution without *rPe*Trxl and DTT. The reaction was initiated by adding 6 μL of 100 mM DTT, and the reaction mixture was incubated at 25 °C for 80 min. Absorbance (Ab) of the precipitate, via insulin reduction, was monitored at 650 nm every 5 min (Microplate Reader: Spectra Max 190; Molecular Devices, Silicon Valley, CA, USA). All samples were used in triplicate; data are presented as mean ± SD. 

#### 4.7.2. ABTS Radical Scavenging Assay

The total antioxidant capacity assay kit, along with the rapid ABTS method (Beyotime, Shanghai, China), was used to determine the ABTS radical (ABTS^+.^) scavenging ability of refolded *rPe*Trxl, according to the manufacturer’s protocol. Briefly, different concentrations (0.1, 0.2, 0.3, 0.4, and 0.5 mg/mL) of refolded *rPe*Trxl diluents were mixed with ABTS working fluid in a 96-well plate. PBS and different concentrations (0.1, 0.2, 0.3, 0.4, and 0.5 mg/mL) of GSH were used as the negative and positive controls, respectively. The same volume of distilled water that replaced the reaction mixture was used as a blank control. Each concentration was measured in triplicate, and the Ab was monitored at 414 nm. The IC_50_ was calculated using SPSS 20.0, and all data are presented as the mean ± SD. The ABTS radical scavenging ability (%) of refolded *rPe*Trxl was calculated as follows:

(1) $$\mathrm{ABTS}\;\mathrm{radical}\;\mathrm{scavenging}\;\mathrm{ability}\;\left(\%\right)=\frac{{\mathrm{Ab}}_{\mathrm{negative}\;\mathrm{control}}-{\mathrm{Ab}}_{\mathrm{sample}}}{{\mathrm{Ab}}_{\mathrm{negative}\;\mathrm{control}}-{\mathrm{Ab}}_{\mathrm{blank}\;\mathrm{control}}}\times 100$$

### 4.8. In Vivo Refolded rPeTrxl Activity Assay 

Injection experiments were performed to determine the effect of refolded *rPe*Trxl in vivo. Experimental groups were exposed to 1/2 Cd − the LC_50_ and treated with an injection of refolded *rPe*Trxl, bovine serum albumin (BSA; Beyotime, Shanghai, China), 50 mM PBS, or no injection; the control group did not receive treatment. The protein injection dose of each individual (2.5–3.5 g) was 20 µg of protein per gram of body weight. After 24 h, three individuals from each group were randomly selected for sampling. Following the collection of coelomic fluid, the ROS Assay Kit (Beyotime, Shanghai, China) was used to detect the relative concentration of ROS using flow cytometry (Becton Dickinson, San Jose, CA, USA), according to the manufacturer’s instructions. The experiment was repeated with three groups of individuals. Data were analyzed using FlowJo_V10 (Ashland, OR, USA) and are presented as the mean ± SD. Statistical analysis (*p* < 0.05) was performed using LSD and Duncan’s post hoc tests in SPSS 20.0.

## 5. Conclusions

In conclusion, we cloned *PeTrxl* gene cDNA from *P. esculenta* and identified *Pe*Trxl as a new member of the Trx superfamily. The protein encoded by the *PeTrxl* gene possesses antioxidant activity. Furthermore, the transcript levels of *PeTrxl* were significantly upregulated in the early stages of Cd stress, and in vivo injection of *rPe*Trxl significantly reduced the levels of intracellular ROS under Cd stress, suggesting that *Pe*Trxl plays an important role in the defense against Cd-induced oxidative stress. Our study provides insight for further research into the role of marine mudflat invertebrate Trxl in Cd stress. Moreover, further studies, including those elucidating the exact function and regulatory mechanism of *Pe*Trxl in maintaining redox homeostasis in *P. esculenta* by gene knockout and determining the secretion pathway of *Pe*Trxl, are necessary.

## Figures and Tables

**Table 1:** Secondary structure of *rPe*Trxl.

Type	Percentage
α-helix	13.5%
β-sheet	39.5%
β-turn	8.9%
Random coil	38.1%

**Table 2:** Primers used in *PeTrxl* cloning, expression, and recombinant analysis.

Primer Name	Primer Sequence (5′–3′)	Description
*PeTrxl*-F1	GCCAGGGTGTATCTGCCAT	PCR
*PeTrxl*-F2	TTATGGTGGAGAAGAGGAGG	PCR
*PeTrxl*-R1	TCTCTTCATCTCCCTGGTTAT	PCR
*PeTrxl*-R2	TGGTTGTGCTAATCGGACT	PCR
3′ *PeTrxl*-F1	CTATTTGTGAGGGATAACCAGGGAG	3′ RACE
3′ *PeTrxl*-F2	CCTTGGACTTGGATAAGGCAGAAG	3′ RACE
5′ *PeTrxl*-R1	CATTGGCCTCCTCTTCTCCACCAT	5′ RACE
5′ *PeTrxl*-R2	TTCACAGCCAGATTTGTTCAGCATG	5′ RACE
*PeTrxl*-F	GCAACAAAGTCAAGGTGGATTC	qPCR
*PeTrxl*-R	CAGCCAGATTTGTTCAGCATG	qPCR
*GAPDH*-F	CCAGAACATCATCCCAGCA	Reference gene
*GAPDH*-R	ACGAACAGGGACACGGAAG	Reference gene
*rPe*Trxl-F	CGC***GGATCC***ATGCCAGTAATTTCCATAG	Recombinant *Pe*Trxl protein
*rPe*Trxl-R	CCG***CTCGAG***GTGTGCTTCCCCCTTT	Recombinant *Pe*Trxl protein

## Data Availability

All of the data generated or analyzed during this study are included in this published article.
